# Volumetric Additive Manufacturing of Dormant Catalytic Chemistries to Generate Silicone Micro‐ and Millifluidic Devices and Instant Molds

**DOI:** 10.1002/advs.202512300

**Published:** 2025-10-16

**Authors:** Johanna A. Vandenbrande, Martin P. De Beer, Erika J. Fong, Aftab Bhanvadia, Massimiliano Ferrucci, Wilson Kong, Daniel Wang, Ryan M. Hensleigh, Michell Marufu, James S. Oakdale, Fangyou Xie, Maxim Shusteff, Johanna J. Schwartz

**Affiliations:** ^1^ Lawrence Livermore National Laboratory 7000 East Ave Livermore CA 94550 USA

**Keywords:** additive manufacturing, casting, instant molds, microfluidics, millifluidics, silicones, volumetric additive manufacturing

## Abstract

Tomographic volumetric additive manufacturing (T‐VAM) rapidly prints solid objects within minutes, accessing photochemistries that are traditionally challenging for layer‐based additive manufacturing methods. This includes high‐viscosity materials, air‐free chemistries, and solid‐state systems. Catalytic chemistries are appealing as a pathway to engineering advanced materials, including tough thermosets, silicone elastomers, and complex block copolymers. However, photoactivated dormant catalytic chemistries, where the catalyst irreversibly activates upon exposure to light, are incompatible with typical tomographic VAM approaches. To address this limitation, a zero‐dose optimization strategy is devised to preserve dormant catalysts in desired regions by keeping them unexposed to light. VAM printed micro‐ and millifluidic devices and instant molds are successfully produced within minutes in silicones polymerized using photoactivated dormant platinum photohydrosilylation catalysts. The printed channels are programmed to be 500  and 2500 µm for the micro‐ and millifluidic devices, and print fidelity is assessed by X‐ray computed tomography. This work demonstrates the potential of zero‐dose optimization to expand the range of chemistries accessible for VAM, enabling the rapid fabrication of complex devices.

## Graphical abstract

We describe use of a novel zero‐dose approach to tomographic volumetric additive manufacturing that has enabled us to print silicone micro‐ and millifluidic devices and instant molds within minutes. The silicones are polymerized using photoactivated dormant platinum photohydrosilylation catalysts that would traditionally be difficult to access in volumetric additive manufacturing.

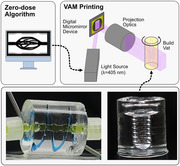

## 1 Introduction

Tomographic volumetric additive manufacturing (T‐VAM), or computed axial lithography (CAL),^[^ ^1^, ^2^, ^3^ ^]^ is an emerging photo‐based additive manufacturing (AM) technique that accelerates the production of 3D objects by irradiating a rotating vat of photoresin with a sequence of angular projections. As VAM removes the need for layer‐by‐layer fabrication, it enables the use of higher‐viscosity resins, air‐free chemistries, stimuli‐responsive materials, and slow polymerization chemistries traditionally incompatible with layer‐based AM methods like stereolithography (SLA).^[^ ^4^ ^]^ For example, T‐VAM printing of step‐growth polymerizations like thiol‐ene chemistries can produce shape memory structures with isotropic shape memory, whereas anisotropies in mechanical response may occur in layer‐based approaches.^[^ ^5^, ^6^ ^]^ In another example, researchers use ring‐opening metathesis polymerization (ROMP) in VAM to cure dicyclopentadiene (DCPD) fully in the solid state.^[^ ^7^ ^]^ This is impractical in layer‐based methods like SLA, which require resin to flow for material recoating during printing.^[^ ^8^, ^9^ ^]^ We are motivated to investigate similar photoactivated catalytic chemistries, since they enable printing of engineering materials with properties not accessible via free‐radical acrylate and thiol‐ene systems. In particular, we seek to make silicones accessible to T‐VAM as silicones are important and versatile materials that possess good biocompatibility, optical transparency, thermal and UV stability, gas permeability, hydrophobicity, and elastomeric mechanical properties. As such, silicones are valuable for a wide range of applications in aerospace, electronics, optics, consumer goods, healthcare, and medical devices.^[^ ^10^, ^11^, ^12^, ^13^ ^]^ However, VAM is particularly challenging with photocatalytic systems with irreversible ligand dissociation such as in silicones, which we refer to as photoactive dormant catalysts, because the activated catalysts continue to react even after the illumination is turned off. This behavior, termed dark cure, severely hampers spatial and temporal reaction control, and thereby shape fidelity and resolution in end use objects.

**Figure 1:**
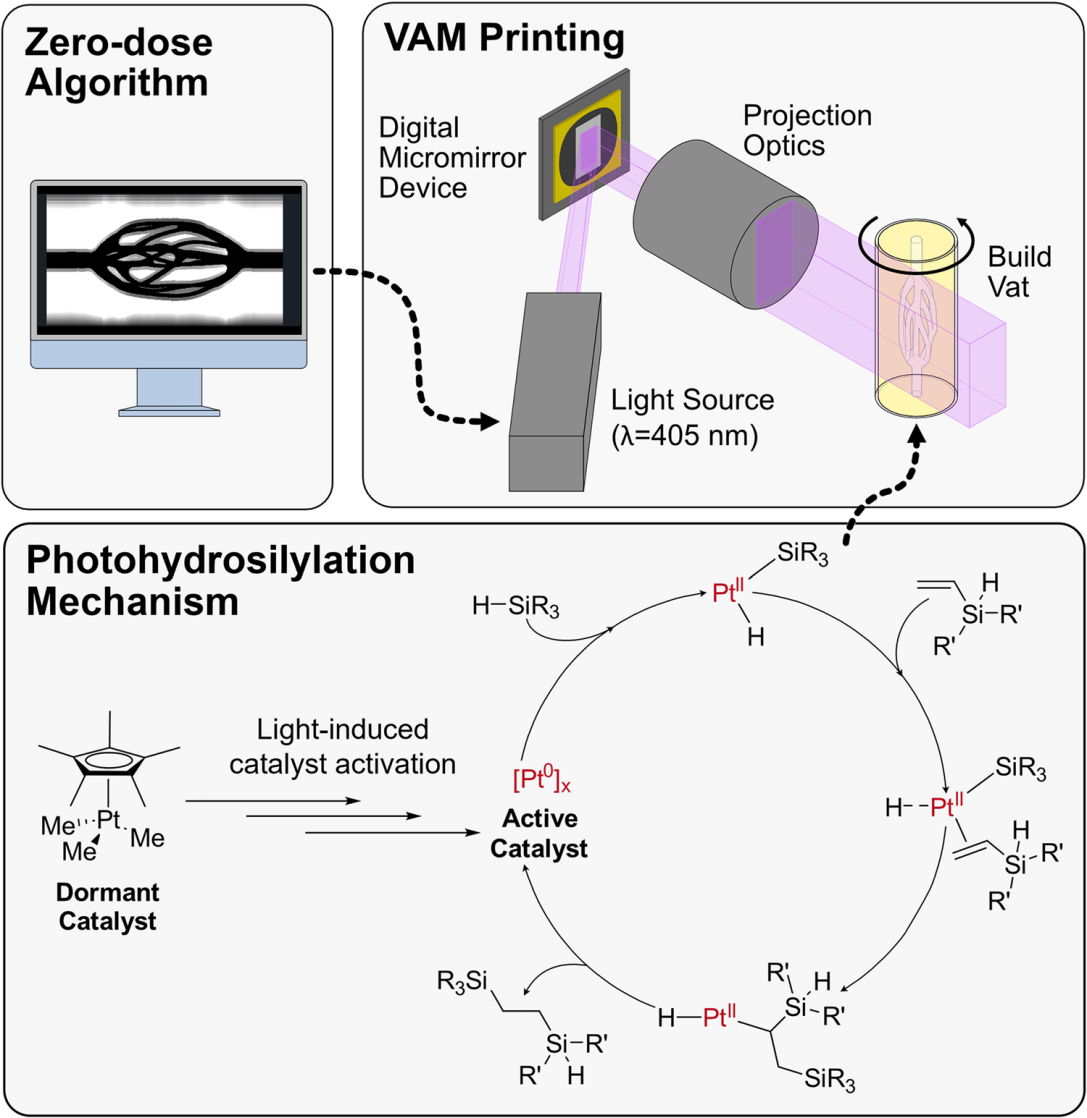
Representative diagram of the VAM printers and the photocatalyzed hydrosilylation chemistry mechanism.

The photohydrosilylation of silicone thermosets uses a photoactive dormant platinum catalyst (**Figure** **1**). Briefly, under light irradiation, the platinum catalyst is deprotected to a series of reactive intermediates and eventually colloidal Pt(0).^[^ ^14^, ^15^ ^]^ This platinum, once exposed, reacts with silane and vinyl precursors, producing crosslinks and forming the polymer network.^[^ ^16^ ^]^ In practice, this means that once any platinum catalyst is activated, it continues to turn over and react until the polymerization becomes diffusion‐limited (for example, the resin cures and solidifies), or the catalyst decomposes. This makes SLA printing of silicones solely through photohydrosilylation difficult, though accessible through specialized equipment that is amenable to high resin viscosities.^[^ ^17^ ^]^ In T‐VAM, it becomes an even larger challenge, as light penetrates the full resin vat. Even the low exposure energy in the out‐of‐part areas can still induce the irreversible dissociation of the platinum dormant catalyst. To enable printing of silicone chemistries, the conventional approach to delivering a distribution of absorbed optical dose in VAM must be modified.

Conventional T‐VAM image generation algorithms, which optimize the projected images for a specific critical dose and assume negligible dark curing, are inadequate in these types of photoactive dormant catalytic chemistries where dark curing is prevalent. After exposure, all exposed voxels begin to polymerize at spatially varying rates dependent on absorbed dose. Extracting the part at the ideal time, such that the desired silicone part is solidified without the surrounding resin being cured, is challenging and inherently timing‐dependent. One method AM researchers use to print silicones instead of photohydrosilylation is to functionalize silicones with acrylate or thiol‐ene groups and photocure via traditional free‐radical polymerization approaches.^[^ ^17^, ^18^, ^19^ ^]^ While these approaches do enable printing of silicones and have reduced dark curing issues, the use of free‐radical polymerization end groups disrupts the polymer network formation. This leads to an inhomogeneous network structure and poor mechanical and aging properties.^[^ ^20^ ^]^ Additionally, acrylates are cytotoxic if not fully reacted, impacting biomedical applications.^[^ ^21^, ^22^ ^]^ To enable printing of objects with ideal silicone material properties, we demonstrate the use of T‐VAM to rapidly create fluidic devices and instant molds within minutes using photohydrosilylation chemistries (Figure 1).

Silicone milli‐ and microfluidic devices are chosen to demonstrate this optimization technique because of their versatile applications, including organs‐on‐a‐chip, soft robotics, and active‐mixing technologies.^[^ ^23^, ^24^, ^25^, ^26^, ^27^, ^28^ ^]^ Typically, silicone fluidic devices are fabricated using soft lithography of polydimethylsiloxane (PDMS), a process that involves replica molding PDMS onto a master mold to create micro‐ or nanopatterned channels on a rigid 2D substrate. Soft lithography is a straightforward process that offers a high degree of accuracy for producing 2D fluidic devices.^[^ ^27^, ^28^, ^29^ ^]^ However, the creation of complex, 3D interconnected channels involves iterative steps, which increase costs, require more labor, and reduce yield. Furthermore, molds for patterning substrates are produced through photolithography, an expensive process often requiring a clean‐room.^[^ ^29^ ^]^ AM has simplified the production of complex, 3D fluidic devices. But in conventional AM methods, like SLA and direct ink write, the layering and filamentation can impact the final mechanical properties, optical transparency, and biological function. For instance, cells may adhere to defects in the fluidic devices.^[^ ^30^ ^]^ In contrast, VAM eliminates the layering effects seen in other AM methods by printing the part all at once. Another advantage of VAM's rapid production time is that casting molds can be made directly from a 3D model, removing the need for an original positive feature.

Herein, we present a novel zero‐dose optimization approach to enable T‐VAM printing of photoactivated dormant catalytic chemistries. This approach ensures no light penetration in desired out‐of‐part regions, thereby eliminating catalyst activation. We target photohydrosilylation chemistries to enable printing of silicone milli‐ and microfluidics for future biological applications including organs‐on‐a‐chip, soft robotics, and biological reactors. We also demonstrated that this technique can instantly produce casting molds by utilizing the zero‐dose optimization strategy. To fully understand the dormant catalytic behavior of our silicone photoresins, we conducted photorheological studies using combinations of catalysts and photosensitizers at varying concentrations and light doses. The results of these studies enable a facile approach to reliably create milli‐ and microfluidic devices within minutes.

## 2 Results and Discussion

### 2.1 Silicone Precursor Resin Composition Design

**Figure 2:**
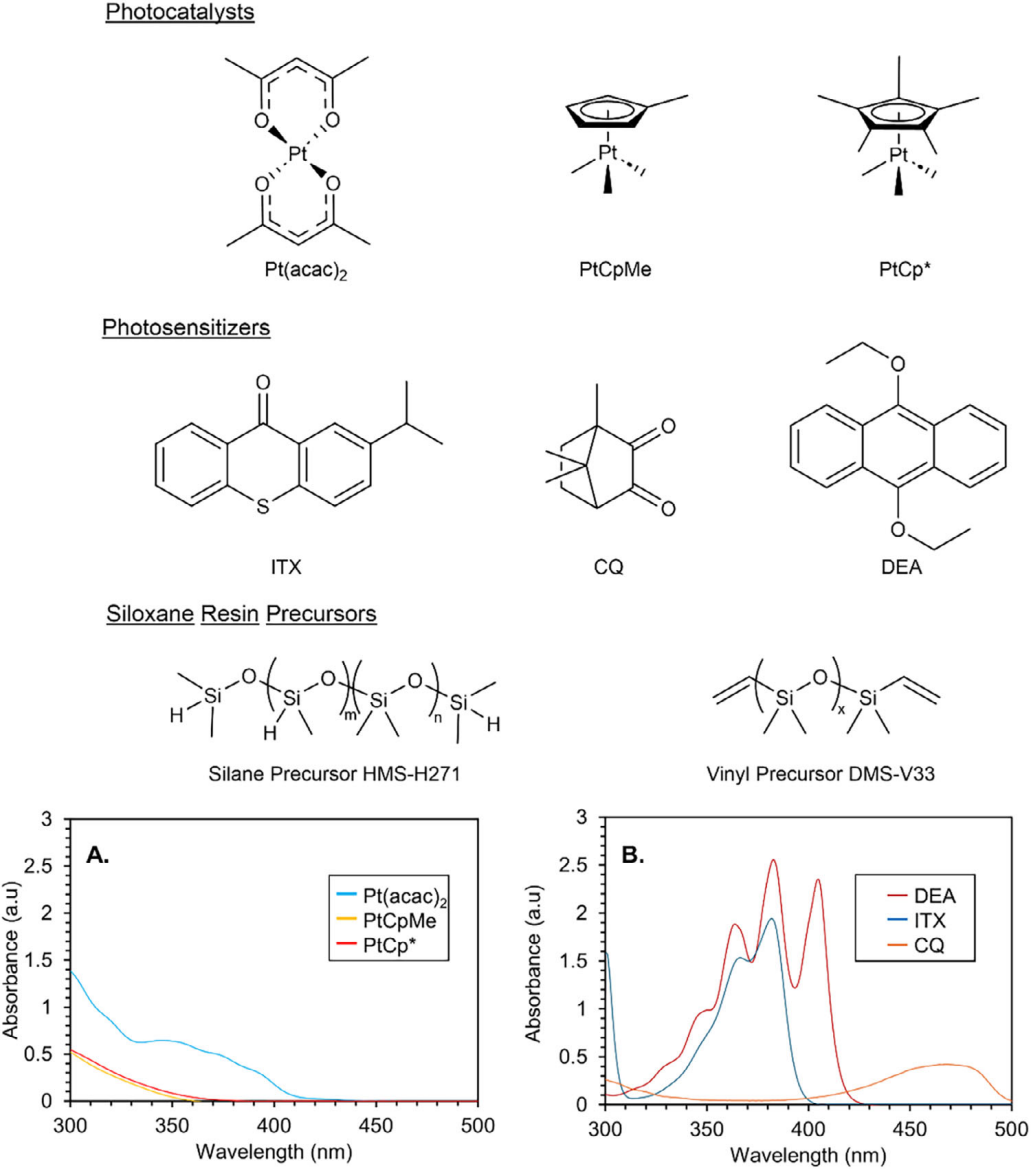
The chemical structures of screened photocatalysts, photosensitizers, and siloxane resin constituents. Refer to the main text for the chemical names for the structures. On the bottom are the UV–vis absorbances of A) photocatalysts, B) photosensitizers. For the UV–vis measurements, solutions of photocatalysts and photosensitizers were prepared in toluene at a concentration of 100 ppm.

To develop silicone photoresins for T‐VAM, we began by identifying and screening three photoactive platinum hydrosilylation catalysts (**Figure** **2**A): platinum (II) acetylacetonate (Pt(acac)_2_), (trimethyl)methylcyclopentadienyl platinum(IV) (PtCpMe), and (trimethyl)pentamethylcyclopentadienyl platinum(IV) (PtCp^*^).^[^ ^31^, ^32^, ^33^ ^]^ The appropriate catalyst loading is determined by establishing a suitable absorbance for the resin. The optimal resin absorbance at the working wavelength, α_*opt*_, that results in the fastest print times for T‐VAM, is related to the printing vial size by α_*opt*_ =  1/*R_vial_*.^[^ ^1^ ^]^

For ⌀ 10 mm and ⌀ 25 mm shell vials, α_*opt*_ is ≈ 0.8 and 1 cm^−1^, respectively. At higher absorbances, insufficient light penetrates the center of the resin vat, hindering part formation. Because photohydrosilylation is a catalytic process, the catalyst remains effective even at low absorption levels, allowing a low exposure dose to trigger the reaction. However with the exception of Pt(acac)_2_, achieving this absorbance with the two other Pt catalysts alone is unfeasible since the species do not absorb at 405 nm, requiring the use of a photosensitizer. To enhance absorbance at 405 nm and boost reactivity during printing, we tested three photosensitizers: 2‐isopropylthioxanthone (ITX), 9,10‐diethoxyanthracene (DEA), and camphorquinone (CQ) (Figure 2B; Table S1, Supporting Information). These photosensitizers were evaluated for their ability to improve light absorption and increase the efficiency of the printing process.

**Figure 3:**
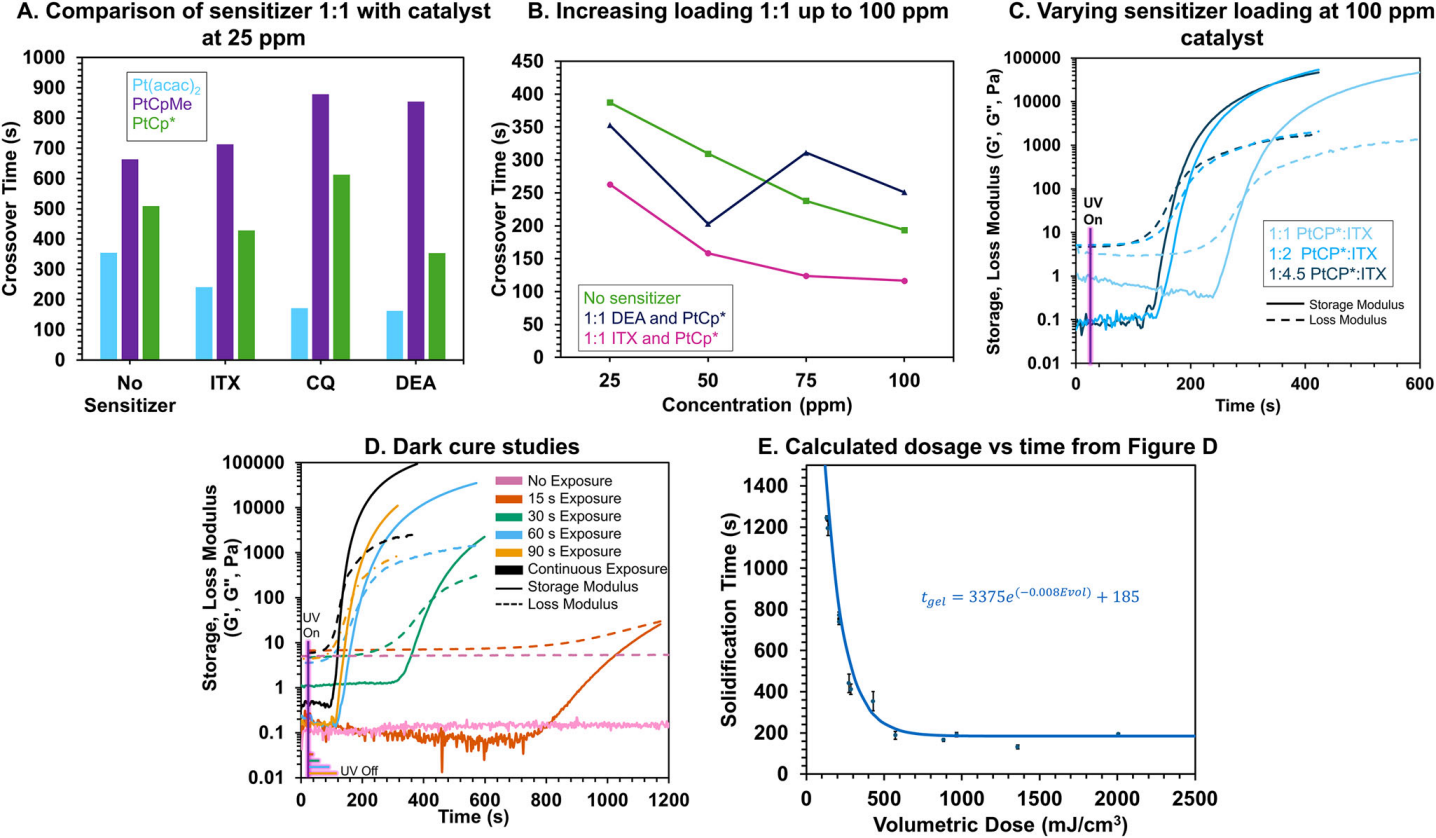
A) Gelation crossover times from photorheological studies of the screened photocatalysts and sensitizers in the siloxane resin under continuous irradiation with 405 nm light. Comparison of 25 ppm of each of the photocatalysts, Pt(acac)_2_,PtCpMe, and PtCp^*^ were each loaded 1:1 on a ppm basis with DEA, ITX, and CQ. B) Increased loading of PtCp^*^ in the resin without photosensitizer, compared to mixing 1:1 with ITX, and with DEA. C) 1:1, 1:2, and 1:4.5 screening of PtCp^*^ with ITX. D) Photorheological dark cure study of 200 ppm ITX and 100 ppm PtCp^*^ at varying exposure times with a 405 nm light source compared to continuous exposure at 10 mW/cm^2^. The doses for the different exposure times are as follows: 15 s = 148 mJ cm^−3^, 30 s = 295 mJ cm^−3^, 60 s = 591 mJ cm^−3^, 90 s = 886 mJ cm^−3^. E) Plot of crossover time versus volumetric energy dose of the 100 ppm of PtCp^*^ and 200 ppm of ITX studies conducted in Figure 3C. Error bars denote standard deviations from replicates in triplicate.

Using photorheology, we compared the crossover times, representing the gelation point, of various catalyst and photosensitizer combinations at a 1:1 ratio. Concentrations ranged from 25  to 100 ppm, under constant irradiation of 20 mW cm^−2^. Among the catalysts, Pt(acac)_2_ exhibited the fastest crossover time (6 min) without the addition of a photosensitizer (**Figure** **3**A). This rapid response is attributed to the catalyst's absorption cutoff at 420 nm, which exceeds the 405 nm wavelength of the light source used. In contrast, the other two catalysts were slower, because of the weaker absorption at 405 nm, with PtCpMe showing the slowest crossover time of 11.2 min. However, without photosensitizers, the photocatalysts alone were too slow for printing using T‐VAM. When maintaining a 1:1 ppm ratio of photosensitizer (CQ, DEA, or ITX), Pt(acac)_2_ exhibited the greatest reduction in crossover time (Figure 3A). Despite this improvement, we observed a noticeable decrease in pot life for the Pt(acac)_2_ formulation under ambient light exposure, leading to its exclusion from further study. Cure times using PtCpMe did not improve with the addition of photosensitizers, so PtCp^*^ was selected for printing. Additionally, CQ generally extended crossover time and was therefore excluded from further photosensitizer screenings at higher concentrations.

With the PtCp^*^ catalyst, we investigated the impact of catalyst and sensitizer loading on cure time. Without photosensitizer, the cure time decreases with increased loading. A maximum loading of 100 ppm was selected as the cutoff. Adding the photosensitizers DEA and ITX at a 1:1 ratio to the catalyst further reduced the crossover time as the loading increased (Figure 3B). We determined that a combination of 100 ppm PtCp^*^ and 200 ppm ITX photosensitizer, corresponding to a 1:2 ratio of catalyst to sensitizer achieved a fast crossover time of 110 s under constant photoirradiation and exhibited a suitable absorbance of 0.093 (Figure 3C). Although this absorbance is lower than the theoretically ideal value for achieving the fastest VAM print rate, the crossover time did not significantly improve with additional ITX. Therefore, further increasing the ITX concentration was deemed unnecessary at this catalyst loading.

A key aspect of process control in T‐VAM fabrication is understanding the relationship of irradiation dose, time, and resulting mechanical integrity (i.e., degree of cure). As with all AM that relies on lithography, the absorbed optical dose is carefully optimized for resolution. In this resin system, notably in contrast to traditional acrylates, an active Pt catalyst will eventually diffuse from the printed structure leading to loss of resolution. Yet premature removal or development of under‐cured parts risks damage to the structure. We conducted photorheological dark cure studies to study this trade‐off, Figure 3D. As expected of the photohydrosilylation mechanism, irradiation times as low as 15 s at 20 mW cm^−2^, or a volumetric dose of roughly 148 mJ cm^−3^, still result in eventual gelation and solidification of the silicone photoresin after 20–30 min. At low doses or short exposure times, the plateau storage modulus is very soft (roughly 100 Pa), which makes handling of the printed object difficult without part deformation. To identify the volumetric dose that is necessary to yield robust parts for extraction, we evaluated when the tan (δ) between the storage and loss modulus decreased below 0.01, which indicates that the object is more solid‐(δ) like and is now mechanically robust enough to endure handling and extraction. Above an absorbed dose of ≈ 500 mJ cm^−3^, we see a plateau in cure time and no further exposure induces a further decrease in tan δ (Figure 3E). Regardless of exposure time, the plateau storage moduli all converged to the same point. This suggests that after 500 mJ cm^−3^, all PtCp^*^ has been fully activated, and light no longer impacts the polymerization. Below 500 mJ cm^−3^, cure times get progressively longer, and small changes in dosage correspond to large changes in cure times anywhere from 5 to 30 min from our photorheological testing. This means that 500 mJ cm^−3^ can be considered the curing threshold dose, with voxels receiving a dose above the threshold effectively curing at the same time, while everything below the threshold will take longer, allowing time for part extraction and cleaning.

### 2.2 Zero‐Dose Optimization Strategy

In order to print structures with hollow features in these silicone materials, we implemented an optimization strategy to generate projections for T‐VAM to keep desired void regions at a zero dose. Conventional error minimization algorithms used to generate projections in VAM do not optimize for high contrast in negative geometries, such as internal voids necessary to create fluid channels. Conventional approaches generally use a thresholding behavior to model gelation, meaning all voxels which receive light energy above a certain critical value will be assumed to be gelled.^[^ ^1^, ^34^, ^35^, ^36^ ^]^ Consequently, the loss function used in typical optimization only reduces the dose delivered to out‐of‐part voxels below a threshold value, but does not completely eliminate it. This approach works well in photopolymerizable materials with a sharp gelation transition (e.g., multifunctional acrylates) but poorly in the silicone material studied here due to the slow transition and significant dark curing. In the photocatalyzed silicone chemistry used here, reliable printing of negative channel features is not possible using standard algorithms without inadvertent curing in the channels.

**Figure 4:**
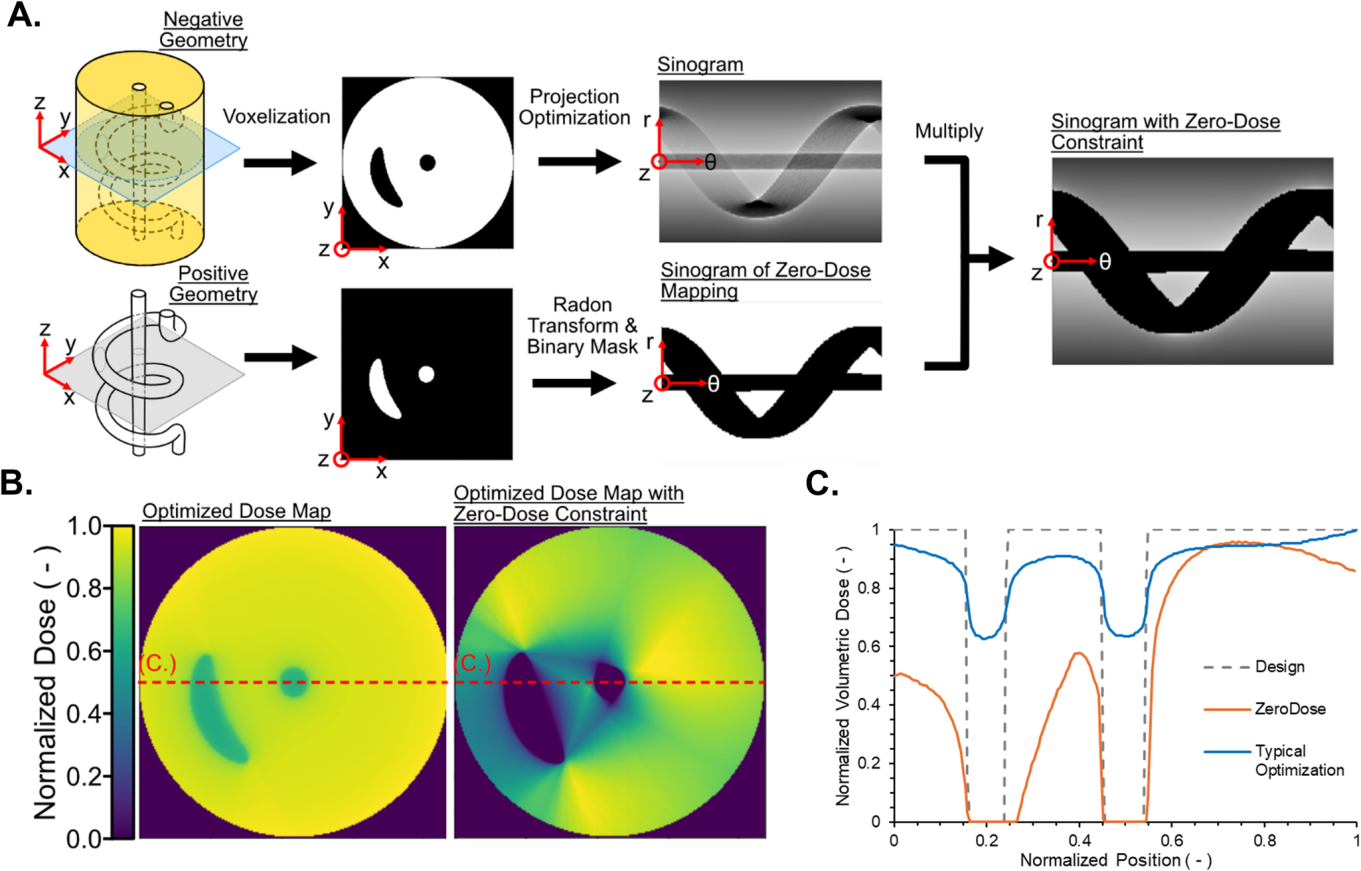
A) Schematic outline for channel masking algorithm to ensure zero in‐channel dose. A binary mask of the forward‐projected positive geometry is multiplied by an optimized sinogram for the negative geometry to give the final “zero dose” projection set. B) 2D z‐slices through the normalized dose map for the optimized projection set and the projection set with the “zero dose” constraint. C) Plot of normalized dose as a function of normalized position for the red dashed line in pane B. As can be seen, using our traditional tomographic optimization (blue line) there is still significant dose absorbed within the desired channel domains (gray dotted lines). Within our zero‐dose approach (orange line) this is removed.

**Figure 5:**
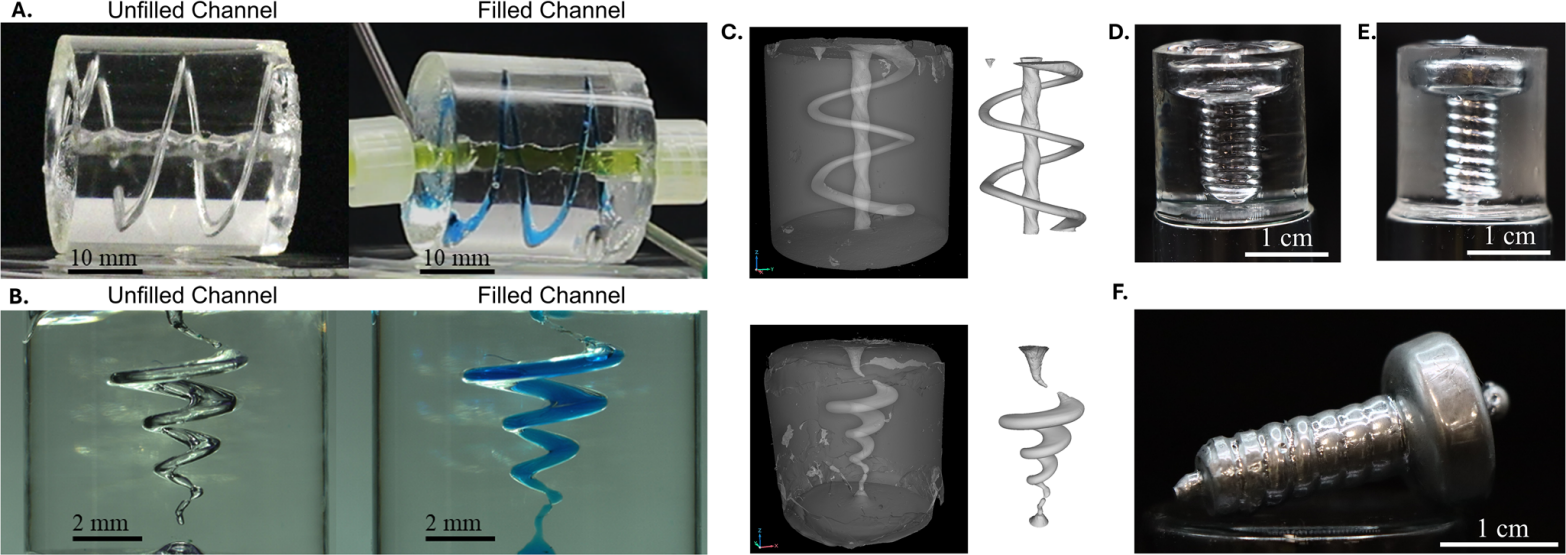
A) T‐VAM printed millifluidic device with and without water dyed with food coloring in the channels (Video S1, Supporting Information). B) Laser‐VAM printed microfluidic device with and without fluid in the channels (Video S2, Supporting Information). C) Images of the CT scans of the millifluidic (top) and the microfluidic (bottom) alongside the channel STLs generated from the CT scans. D) T‐VAM printed instant mold with a screw void in the center. E) Instant mold filled with gallium metal. F) Gallium screw extracted from the instant mold.

Our zero‐dose approach, shown in **Figure** **4**A, generates an initial projection set using a typical minimization approach^[^ ^1^, ^35^, ^36^, ^37^ ^]^ and a mask using the Radon transform of the positive channel geometry to define the channel void. The mask is then multiplied with the initial projection set to force all pixels that would deliver light into the channel to zero. The effect of forcing exposure into the channels to zero on the dose map is shown in Figure 4B. Figure 4C shows the relative dose as a function of the normalized position along the red dotted line seen in Figure 4B. Figure 4C shows clearly that the traditional approach achieves a minimum negative feature (i.e., in‐channel) dose of ∼0.6 normalized dose for the example geometry, but our zero dose approach sets these minima to zero, at the expense of decreasing the overall dose. The drawback of the zero‐dose approach is that it introduces geometric errors in concave regions when they are occluded from all angles swept during the circular projection path during printing. This can be seen clearly in printed parts as shown in **Figure** **5** and will be discussed further in Section 2.3.

Because illumination image sets calculated by standard tomographic optimization approaches irradiate the entirety of the resin vat, this results in a race against time to keep fluidic channels clear and prevent outgrowth. An example of this less effective timing‐based approach for a part can be found in Figures S1 and S2 (Supporting Information). Forcing the in‐part exposures to zero eliminates all dose delivered into the voids, significantly enhancing contrast between positions inside and outside the part. This fundamentally makes printing of silicone fluidics and casting molds possible, enabling facile printing of voids in minutes without worry of photocatalyst activation and curing within the out‐of‐part regions. By using a zero‐dose approach, we can avoid this timing problem and maintain object fidelity. To verify this, we created a negative channel graded cylinder mold, with progressively decreasing channel width, to see the minimum viable channel diameter in the zero‐dose approach for the LED‐based T‐VAM (Figure S3, Supporting Information). Within this test, the void space was infilled with paraffin wax, and the diameters of the wax structures were measured to compare to the programmed void channel dimensions. In this case, we find that the structures showed good channel fidelity above 0.89 mm. At 0.89 mm, the channel was roughly two thirds the programmed size, suggesting overcure into the void domains (Table S2, Supporting Information). Below 0.89 mm, no channels were formed. Future efforts will be needed to improve the resolution of the negative feature domains to enable more complex fabrication and enhanced part fidelity.

### 2.3 T‐VAM of Fluidic Devices and Instant Molds Using a Zero‐Dose Approach

Using the optimized resin formulation, we printed three fluidic devices and two casting molds using two different T‐VAM printers that employ different 405 nm light sources. The differences between the T‐VAM printers are detailed in Table S3 (Supporting Information). Millifluidic parts were printed on a T‐VAM system that uses an LED‐coupled DLP projector with a maximum intensity of 58 mW cm^−2^ (Figure 5A; Figure S4, Supporting Information). A microfluidic device was printed on a laser‐based T‐VAM system with a maximum intensity of 1000 mW cm^−2^ (Figure 5B). The laser‐based T‐VAM system was utilized to achieve higher resolution (≈5.4 µm pixel size) compared to the commercial DLP projector (≈14.5 µm pixel size).

During the printing process, the refractive index change of the cured silicone is essentially indistinguishable from the uncured resin by eye or shadowgraph, changing from 1.44 (liquid) to 1.45 (solid) at 405 nm. This makes identifying when the print is formed difficult, as depicted in the before and after photos in the Figure S5 (Supporting Information), and instead we rely on timing from our photorheological dark cure study.

As described in section 2.2, our rheological investigation indicated that resin exposed above 500 mJ cm^−3^ cures at roughly the same time, with a crossover time of ≈ 150 s or a plateau time of ≈ 300 s, regardless of continued exposure. Using our zero‐dose approach, we target this dose for all curing voxels, irradiating the resin and waiting for sufficient time for the fluidic positive regions to dark cure and solidify. By maintaining zero dose in the negative channels, they remain liquid throughout this wait time and processing. In general, we found that our timing approach based on the dark cure studies was robust, and parts could be extracted after 4–6 min of waiting after exposing all positive voxels to a volumetric dose of ≈ 500 mJ cm^−3^. This enabled all the irradiated regions to cure, leaving the unexposed negative channels uncured, after which the parts were extracted, and the channels solvent‐rinsed and tested with dyed water. The instant molds were produced using the same method for fabricating the millifluidic device. After irradiation in the T‐VAM printer and dark curing, we removed excess uncured resin from the void region (Figure 5D; Figure S6A, Supporting Information) and filled it with melted gallium (Figure 5E). Once the gallium solidified, we extracted the part from the mold, as shown in Figure 5F and Figure S6B (Supporting Information). Minor defects in the cast molds resulted from air pockets trapped during the filling process.

The millifluidic and microfluidic channels, as seen in Figure 5, were printed in minutes from silicone material that exhibited lower elongations to break than molded samples (averaging 48% strain vs 110% respectively) (see Figure S7 and Table S4, Supporting Information), but did maintain similar elastomeric behavior. The reduced elongation at break and ultimate tensile strength observed in the printed dogbones, compared to the casted dogbones, are likely due to the unintended material outgrowth beyond the desired geometry in the necking region during printing, as well as potential deformation in this area during extraction. Because of the quick cure times and activated catalyst within the irradiated parts of the vat, the printed dogbones had to be rapidly removed from the resin to prevent unwanted curing of material outside of the desired geometry. This quick extraction of the soft silicone part from the viscous resin can also deform the part.

**Figure 6:**
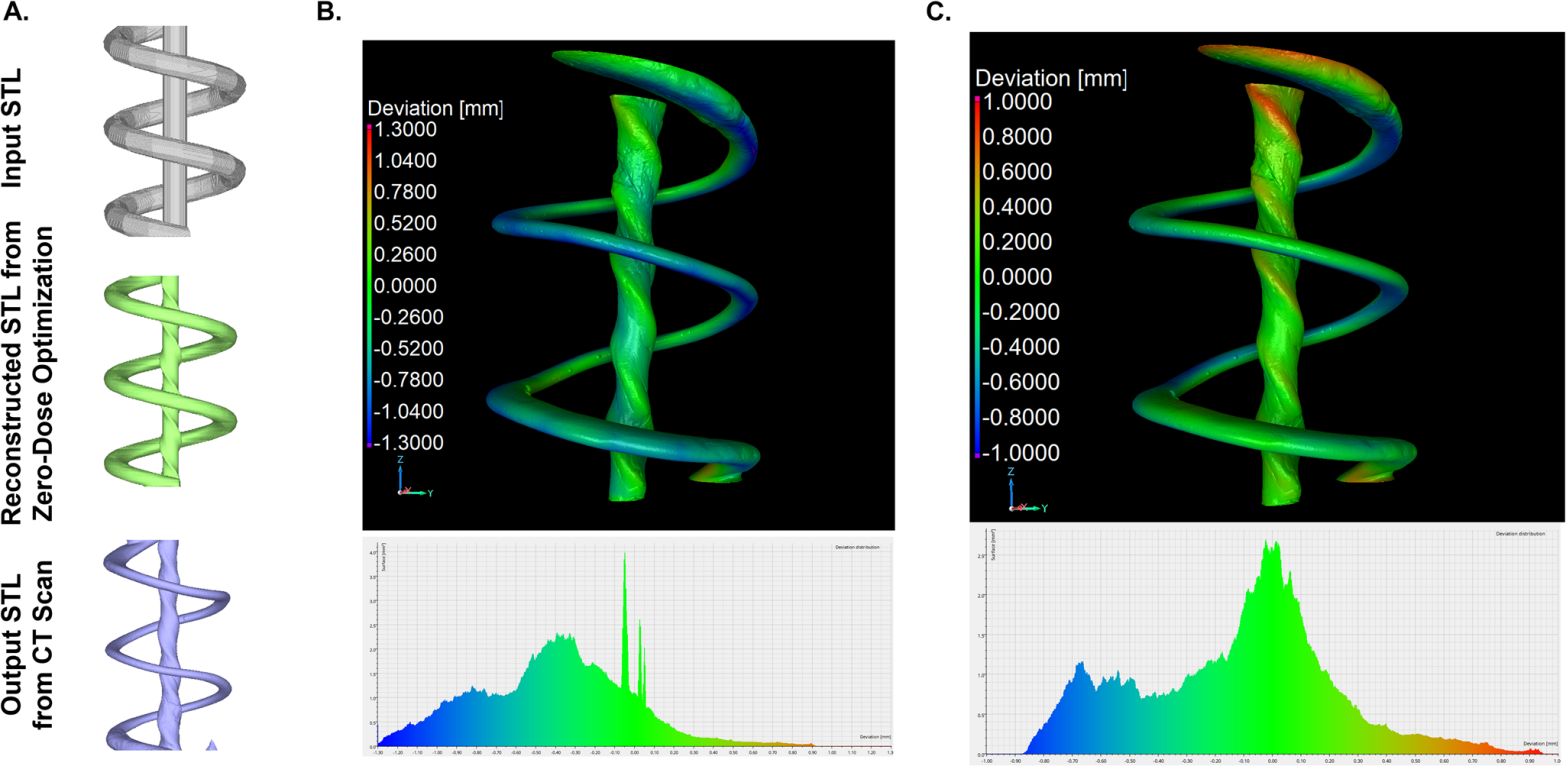
A) From top to bottom, the 1) Target geometry, 2) Reconstructed isosurface from zero‐dose optimization, and 3) Printed geometry generated from the CT scan of the VAM printed millifluidic device. B) Deviation map comparing the CT scan of the printed structure to the input target geometry. C) Deviation map comparing the CT scan of the printed structure to the zero‐dose reconstruction.

It became apparent during the printing of the fluidic devices that there was a difference between the original input STL and the output printed geometries. For example, the central channel of the printed millifluidic device (**Figure** **6**A–C) clearly shows spiraling central channel features whereas the desired input geometry has a straight‐walled center channel. To better understand this, we conducted X‐ray computed tomography (CT) scans on the printed parts (Figure 5C) for quantitative comparison with the input geometries. To determine whether the discrepancy originates from our zero‐dose optimization process, we also reconstructed STL‐format geometries corresponding to surfaces of constant dose exposures from the optimization generated dose maps. For the millifluidic device, the geometry of the printed channel in the CT scan more closely resembles the reconstruction isosurface produced by the zero‐dose optimization than the programmed geometry. This can be seen in Figure 6B, where the printed structure was on average −0.40 mm smaller than the input geometry, with a standard deviation of 0.38 mm from the mean. In contrast, comparing the reconstruction zero‐dose optimization dose map output to the printed millifluidic part, shows a much smaller deviation (Figure 6C), with an average of −0.13 mm smaller and a standard deviation from the mean of 0.34 mm. The Radon transform operation causes shadowing from the surrounding spiral that distorts the central channel, during dose reconstruction, causing a spiral pattern.

Relative to the millifluidic part, the printed microfluidic spiral channel exhibited a greater deviation from both the input geometry and the dose‐reconstruction STL from the optimization. This can be seen in Figures S8 and S9 (Supporting Information), where the CT scan of the printed channel is noticeably smaller than input or reconstructed STL. The dose‐reconstruction STL from the optimization shows the channels are compressed along the X and Y axes, and these effects are visible in the output print CT. The inaccuracy in the zero‐dose optimization compared to the input geometry likely arises because the zero‐dose masking step is imposed on an image‐set optimized with a cost function that is not aware of the zero‐dose step. Consequently, creating deviation maps for the microfluidic device was not possible due to the extent of shrinkage. In areas where the channels are larger, such as the top and bottom of the millifluidic outer channel, this is likely due to the cleaning and processing protocol. To fully remove uncured resin, isopropyl alcohol and high‐pressure air were pushed through the channels. In the future, improving the optimization to maximize input shape fidelity and real‐time feedback and monitoring into the T‐VAM print is expected to improve print agreement and repeatability.^[^ ^38^ ^]^ Further side‐by‐side comparisons of the STLs for the micro‐ and millifluidic and the screw instant cast can be found in Figures S7–S11 (Supporting Information).

## 3 Conclusion

In this study, we demonstrate T‐VAM of a dormant photohydrosilylation catalytic system to produce silicone micro‐ and millifluidic devices. Using a zero‐dose optimization strategy, this T‐VAM approach could reduce the time and the cost associated with producing 3D fluidic devices. This zero‐dose approach enables T‐VAM to access dormant, dark‐curing photocatalytic chemistries, such as photohydrosilylation, and broadens the range of printable materials. Using photorheology, we identified the optimal photocatalyst‐photosensitizer combination to achieve rapid crossover times suitable for T‐VAM. We printed multiple fluidic structures and instant molds, then used X‐ray CT scans to visualize the internal channels to identify how the printed channels differ compared to the targeted geometries. Future efforts will focus on stabilizing the silicone resin using photocatalyst inhibitors or quenching agents and improving the mechanical properties of the resulting silicone materials by incorporating fillers. Further investigations into tomographic optimization strategies are expected to reduce barriers to using catalytic chemistries and engineering materials in T‐VAM. Ultimately, we have enabled printing of negative features in a way that was not previously possible in VAM.

## 4 Experimental Section

### Materials and General Considerations

Vinyl precursor DMS‐V33 (vinyl terminated polydimethylsiloxane, 3500 cSt) and silane precursor HMS‐H271 ((25%–30% methylhydrosiloxane) – dimethylsiloxane copolymer, hydride terminated, 24–60 cSt) were purchased from Gelest. Platinum catalysts (Trimethyl)pentamethylcyclopentadienyl platinum(IV) (PtCp^*^) and (Trimethyl)methylcyclopentadienyl platinum(IV) (PtCpMe) were obtained from Strem. Platinum (II) acetylacetonate (Pt(acac)_2_), camphorquinone (CQ), and 9,10 diethoxyanthracene (DEA), as well as toluene, isopropyl alcohol, hexanes, and acetone were obtained from Sigma–Aldrich. 2‐Isopropylthioxanthone (Speedcure, ITX) was acquired from Sartomer. Clear V4 resin and Form3 printers were acquired from Formlabs. Wax paraffin was acquired from Spectrum Chemical MFG Corp. All materials were used as received without further purification. Rheological and tensile tests were conducted using TA DHR‐2 rheometer. Light intensities were measured using an Omnicure 2000(Excelitas). The UV–vis was measured usingeither a UV1900 (Shimadzu) or a Cary 5000 (Agilent) with a 1 cm plastic cuvette. For the UV–vis measurements, solutions of photocatalysts and photosensitizers were prepared in toluene at a concentration of 100 ppm.

### General Resin Preparation

DMS‐V33 (0.077 mmol functional group/gram vinyl precursor) and HMS‐H271 (4.2 mmol functional group/gram silane precursor) precursors were weighed and added to achieve a functional group ratio of 1:1.7 vinyl to silane, respectively. This ratio was chosen to create a reactive silicone resin comparable to industry standard systems.^[^ ^39^ ^]^ Platinum catalyst (PtCp^*^, PtCpMe, or Pt(acac)_2_) was added at a concentration of 25, 50, 75, or 100 ppm (calculated w/w) of total precursors, from a 1 mg mL^−1^ stock solution in toluene (**Table** **1**). Photosensitizer (ITX, CQ, or DEA) was added to be either a 1:1, 2:1, or 4.5:1 ppm ratio of the catalyst from a 10 mg mL^−1^ stock solution in toluene. This means for the entirety of the study, the range of toluene incorporated was from 0.028 to 145 uL per gram of silicone resin. For printing, the ratio is 120 µL per gram of silicone resin, (including both DMS‐V33 and HMS‐H271) or roughly 10 vol %.

**Table 1:** Molar equivalent loading based on 1 g of DMS/HMS resin, and corresponding 1 or 10 mg mL^−1^ stock solutions in toluene.

	25 ppm	50 ppm	75 ppm	100 ppm
ITX (10 mg mL^−1^)	98 nmol	197 nmol	295 nmol	393 nmol
CQ (10 mg mL^−1^)	150 nmol	300 nmol	451 nmol	602 nmol
DEA (10 mg mL^−1^)	94 nmol	188 nmol	282 nmol	376 nmol
PtCp^*^ (1 mg ml^−1^)	67 nmol	133 nmol	200 nmol	266 nmol
PtCpMe (1 mg ml^−1^)	78 nmol	156 nmol	235 nmol	313 nmol
Pt(acac)_2_ (1 mg ml^‐^^1^)	64 nmol	127 nmol	191 nmol	254 nmol

All components were mixed three times at 2000 rpm for 1 min in a Planetary Centrifugal Mixer ARE‐310(Thinky). Multiple short mixes were used to prevent heating, and the mixed resin was stored in a cool, dark location until ready to use. Aluminum foil around the container was also used to limit ambient light‐resin interactions. Ultimately, a resin formulation with 100 ppm PtCp^*^ and 200 ppm ITX was chosen for VAM printing. An example resin formulation used for printing is as follows: 40 g of DMS‐V33, 1.2 g of HMS‐H271, 4 mL (11 µmol) PtCp^*^ solution, and 0.8 mL (31 µmol) ITX solution.

### Photorheological Analysis

Absorption coefficients at 405 nm were identified for each resin using a UV–vis spectrophotometer (UV‐1900) as described previously.^[^ ^40^ ^]^ Gelation kinetics were determined using a rheometer (DHR‐2), with resin placed between an aluminum upper plate and acrylic or quartz lower plate at a gap of 200 µm. Aluminum, acrylic, and quartz plates were cleaned before use, through 1–2 min of sonication in isopropyl alcohol, as the as‐received disposable plates potentially have machining oil or other residue that inhibits silicone curing. Measurements were recorded at an oscillation frequency of 2.5 rad s^−1^ with a strain of 3% applied to the resin. After 30 s of baseline measurement, the light was turned on for either constant illumination or dark cure studies. Samples were exposed to illumination from an arc lamp with a 405 nm filter, ranging in intensity from 5 to 20 mW cm^−2^ (Omnicure 2000). For dark cure studies, the illumination was only for a short period of time, ranging generally from 15 to 60 s of exposure, and the measurement continued in the dark until gelation was achieved, or up to 20 min.

### LED‐Based Tomographic Volumetric Additive Manufacturing of Millifluidics

Readers may find information about volumetric dose calculations from previously published literature.^[^ ^33^, ^36^ ^]^ VAM prints were performed in a custom printer setup equipped with a 405 nm LED light engine (3DLP9000, Digital Light Innovations), with a telecentric lens and maximum intensity of 58 mW cm^−2^ at the surface of the resin vial. Resin was poured into one‐inch diameter vials, warmed to ambient room temperature, and degassed. Vials were inserted into the rotation stage (HDR50, ThorLabs) and the rotation rate of the vial was set to 10 degrees per second. Vials were lowered into a silicone fluid (DMS V33) index matching bath. The vials were exposed for 3 rotations for a total dose of over 500 mJ cm^−3^ (estimated dose 540 mJ cm^−3^). This dose, as shown in Figure 3 and Figure S2 (Supporting Information), was determined to be the critical limit to consistent cure times. Following exposure, parts remained in the vial for 4–5 min, (roughly 400 s total time including exposure) before extraction. Extracted parts were washed in isopropyl alcohol and hexanes solutions.

The printed millifluidic devices and instant molds were photographed using a Canon EOS Rebel T8i camera.

### Laser‐Based Tomographic Volumetric Additive Manufacturing of Microfluidics

The microfluidic devices were printed with a custom laser‐based VAM system utilizing a 405 nm laser (5 W max power) as the light source incident on the DLP9500 DMD (Vialux) surface. This system utilized a custom designed 4F telecentric lens system to achieve ≈0.5x magnification, resulting in ≈5.4 µm pixel size projected at the focal plane with an image area of ≈10.368 mm x 5.832 mm. During the print, the maximum intensity at the focal plane was ≈ 32 mW cm^−2^. Resin was poured into 10 mm diameter NMR tubes (New Era Precision NMR Sample Tubes), warmed to room temperature, and degassed. Vials were inserted into the rotation stage (Zaber X‐RSB060AD‐E01) and the rotation rate of the vial was set to 60 degrees per second. Vials were lowered into a silicone fluid (DMS V33) index matching bath. The vials were exposed for 10 rotations for a total dose of over 500 mJ cm^−3^ (estimated dose 600 mJ cm^−3^). To improve the ease of removing uncured resin from the printed part, adherence of the printed part to the interior walls of the NMR tube was ensured by further irradiating the resin close to the tube edge with a thin rectangle of light 1 min at 60 deg s^−1^. Following exposure, parts remained in the vial for 6.5 min (roughly 510 s total time including exposure), before extraction. Extracted parts were washed in isopropyl alcohol and hexanes solutions and uncured resin was blown out using pressurized air.

The printed microfluidic device was photographed using a Keyence microscope VHX X1, and a Canon EOS Rebel T8i camera.

### Zero‐Dose File Generation

Target geometries were defined as solid cylinders the size of the vial with void spaces defined by channel diameters of 2500 or 500 µm (SolidWorks). This geometry was voxelized based on the resolution of the printer; regions to be polymerized are 1, void regions are 0, *f_T_*. Initial projections were generated using typical VAM algorithms.^[^ ^1^, ^34^, ^35^, ^36^ ^]^ To identify pixels in the projections which contribute to dose in the void regions, the target geometry was inverted to generate a “void geometry,” $f_{T_{void}}$, where the channels are 1 and the rest is 0. This void geometry was projected using the Radon transform, $P(f_{T_{void}})$; regions greater than 0 indicate pixels which contribute to dose in the intended void region. This is used to mask the projection, by setting any pixels which are greater than 0 in $P(f_{T_{void}})$ to 0 (Figure 4). The resulting masked projection is back projected using the attenuated Radon transform to generate the estimated dose distribution. For each slice in Z (height dimension), the intensity of the projection is scaled to deliver a specified average dose (critical dose) at the boundary of the void and gel regions. The target dose at the boundary can be adjusted to ensure the experimental print times are reasonable.

### Generating STLs Based on the Dose Maps Generated by the Zero‐Dose Optimization

To estimate the print geometry based on the reconstruction dose, the critical dose used to generate the projection set (average dose at the boundary of void and gel regions) was used to threshold the reconstruction dose volume. To obtain the channel geometry, the RGB levels were inverted using an invert LUT to ultimately threshold for the interior of the negative channel domains. Using the 3D viewer plugin, the threshold was calculated using the equation below. The surface was displayed, threshold was entered based on the equation, and the resampling was set at 2 to generate the 3D model (FIJI, ImageJ) The resulting STL is then scaled to match the height of the target model.

(1) $$\begin{array}{ccc} \mathrm{Threshold} & = & \left(\frac{\mathrm{Original}\;\mathrm{Maximum}\;\mathrm{Pixel}\;\mathrm{Value}}{\mathrm{Maximum}\;8\;\mathrm{Bit}\;\mathrm{Pixel}\;\mathrm{Value}}\right)\\ & & \times \left(\mathrm{Original}\;\mathrm{Maximum}\;\mathrm{Pixel}\;\mathrm{Value}-1\right) \end{array}$$

### Tensile Samples and Testing

Two sets of samples were prepared for tensile testing. ASTM D638 type IV dogbones were used as the tensile samples, at 90% size, to accommodate the maximum working print size within the VAM setup. Samples were made through volumetric printing and through bulk casting. For casted parts, dogbone molds were fabricated in a Form3 printer. Molds were washed in isopropyl alcohol and cured within a FormLabs FormCure prior to use. Resin was poured into the molds, degassed, and covered with an acetate sheet. Resin was then cured in a light box (spdiUV UV exposure lab chamber at 18 mW cm^−2^) for 20 min and physically removed from the molds.

Tensile testing was performed on a rheometer using a tensile fixture. Dimensions of the dogbones were recorded and samples were secured within the grips. Testing was conducted at a constant linear rate of 1 mm min^−1^, and the modulus at 10% strain of the resin was determined for both cast and volumetric printed parts for comparison.

### Fluidic Testing

Silicone tubing with plastic inlet adapters were physically embedded into VAM printed fluidic channels. Syringes filled with dyed water were attached to the other ends of the tubing and used to push fluid through the channels. Videos and pictures of fluid flow through the channels were taken using a Canon EOS Rebel T8i camera.

### Wax Mold Resolution Analysis

Wax paraffin was completely melted in an oven at 60 °C, and the printed mold was preheated to the same temperature. The molten wax was then injected into the mold cavity. After cooling to room temperature, the solidified wax part was carefully removed from the silicone mold. The wax part was subsequently photographed using a Keyence VHX X1 microscope and a Canon EOS Rebel T8i camera.

### Liquid Metal Casting

Gallium metal shots (Indium Corporation; 99.9999%) were melted in a glass vial at 100 °C on a hot plate. The liquid gallium was then drawn into a syringe and injected into the VAM printed overhead inlet channel while blocking the channel outlet with a glass slide. The metal was allowed to cool and solidify in a freezer prior to extraction from the VAM silicone mold. Several channel shapes were investigated to examine the conformability of liquid gallium with the VAM printed silicone. The gallium part was photographed using a Canon EOS Rebel T8i camera.

### X‐Ray Computed Tomography (CT) Scans

CT data was acquired on a Zeiss Xradia 510 Versa using a flat panel detector comprising 3072 (horizontal) × 1944 (vertical) square pixels with side lengths of 74.8 µm. The acquisition settings are provided in Table S4 (Supporting Information). Dynamic ring reduction (DRR) was enabled in both acquisitions; DRR applies random shifts of the sample at each projection to reduce ring artifacts in the CT data. To minimize cone‐beam artifacts, each sample was tilted such that its cylindrical axis was offset by the axis of rotation by ≈ 30°. Tomographic reconstruction was performed using the implementation of filtered backprojection in Zeiss Reconstructor and with the settings provided in Table S5 (Supporting Information).

The CT data was loaded onto VGStudio MAX 2024.4. Advanced gradient‐based surface determination was applied from an initial isosurface defined at an attenuation gray value 50% between the gray value of the sample material and background, i.e., air. Input and Reconstruction STL files were imported and registered to the CT determined surfaces using a best‐fit approach. Nominal‐actual comparison was applied to determine surface deviations between the CT surfaces and each STL file.

### Channel Size Comparisons

To compare the channel dimensions of millifluidic devices (Figure S8, Supporting Information) and microfluidic devices (Figure S9, Supporting Information), the corresponding STLs were uploaded to Meshmixer. The measure function in the analysis tab was used to determine channel diameters along the x‐axis and thicknesses along the y‐axis. Multiple points within the fluidic devices were analyzed, and the average values, along with their standard deviations, were calculated.

## Conflict of Interest

The authors declare no conflict of interest.

## Author Contributions

J.A.V. wrote the manuscript and printed the microfluidic devices, instant molds, and characterized the structures. M.P.D.B. conducted preliminary formulations testing and photorheological studies and contributed with E.J.F. to the zero‐dose optimization approach. A. B. helped with the laser VAM printing and projection. D.W. conducted preliminary formulations testing, photorheological studies, and tensile testing of silicones. R.H. and M.M. assisted in millifluidic device printing. F. M. conducted the CT scans of the fluidic devices and compared the comparisons of the CT images and the STLs made from the tomographic dose maps. The tomographic dose maps were formed by J.A.V. with help from A.B. and E.J.F.W.K. provided the gallium metal used and infilled the printed casts. J.S.O. and F.X. assisted with preliminary chemistries and manuscript editing. M.S. and all authors contributed to the manuscript editing. J.J.S. conducted and oversaw initial formulations and printing results, as well as writing and finalizing this effort.

## Supporting information



Supporting Information

Supporting Information

Supporting Information

## Data Availability

The data that support the findings of this study are available from the corresponding author upon reasonable request.
