# Supplementary material for: Volumetric Additive Manufacturing of Dormant Catalytic Chemistries to Generate Silicone Micro‐ and Millifluidic Devices and Instant Molds
Source: Adv Sci (Weinh). 2025 Oct 16;13(7):e12300. doi: 10.1002/advs.202512300 (PMC12866854; doi:10.1002/advs.202512300)
Supplement: Supplementary file 1 — Supporting Information [file ADVS-13-e12300-s002.docx]

Supplemental Information

**Volumetric Additive Manufacturing of Dormant Catalytic Chemistries to Generate Silicone Micro- and Millifluidic Devices and Instant Molds**

*Johanna A. Vandenbrande, Martin Patrick De Beer, Erika Jo Fong, Aftab Bhanvadia, Massimiliano Ferrucci, Wilson Kong, Daniel Wang, Ryan Michael Hensleigh, Michell Marufu, James Spencer Oakdale, Fangyou Xie, Maxim Shusteff, and Johanna J. Schwartz*.*

| Compound | Absorbance at 405 nm, 100 ppm |
| --- | --- |
| DEA | 2.4 |
| ITX | 0.0044 |
| CQ | 0.077 |
| Pt(acac)_2_ | 0.10 |
| PtCpMe | 0.0028 |
| PtCp* | 0.0019 |

**Table S1.** UV-Vis absorption at 405 nm, 1 cm path length, for 100 ppm of the photosensitizer or photocatalyst in the silicone resin.


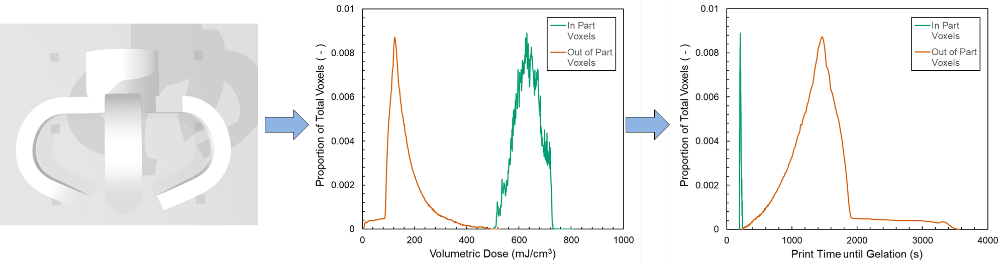


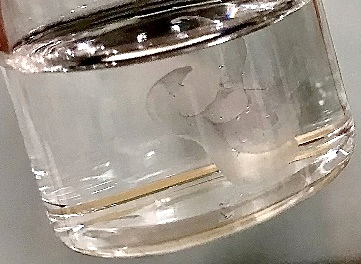

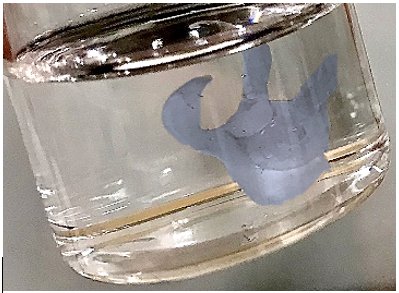


**Figure S1.** Representative schematic of timing control method for positive part VAM printing and extraction, in a four-arm gripper design. Using our photorheological dark cure studies, dosage is converted to time-to-extraction for in-part (green) and out-of-part (orange) voxels. Resulting picture is of printed silicone gripper extracted after 108 s exposure for a target dosage of 540 mJ cm^-3^ and a 5 min wait time (total time, 408 s). The semitransparent gripper is colored blue through PowerPoint photo-formatting in the final image to enhance visualization.

**Figure S2.** Representative impact of wait time on outgrowth in photohydrosilylation polymerization of 1.5 mm silicone dot tests with a 5-min exposure time (with varying intensities) and a 10 min wait time (total of 900s cure time). Circles represent RGB pixel intensities of dots that correspond to estimated volumetric energy dosage experienced by dots at the center of the resin container in our 100 ppm PtCP* resin with 200 ppm ITX. Below 126 RGB no solid dots were formed. Above 126 RGB dots exhibited outgrowth along the path of exposure resulting in oblong disc shapes.


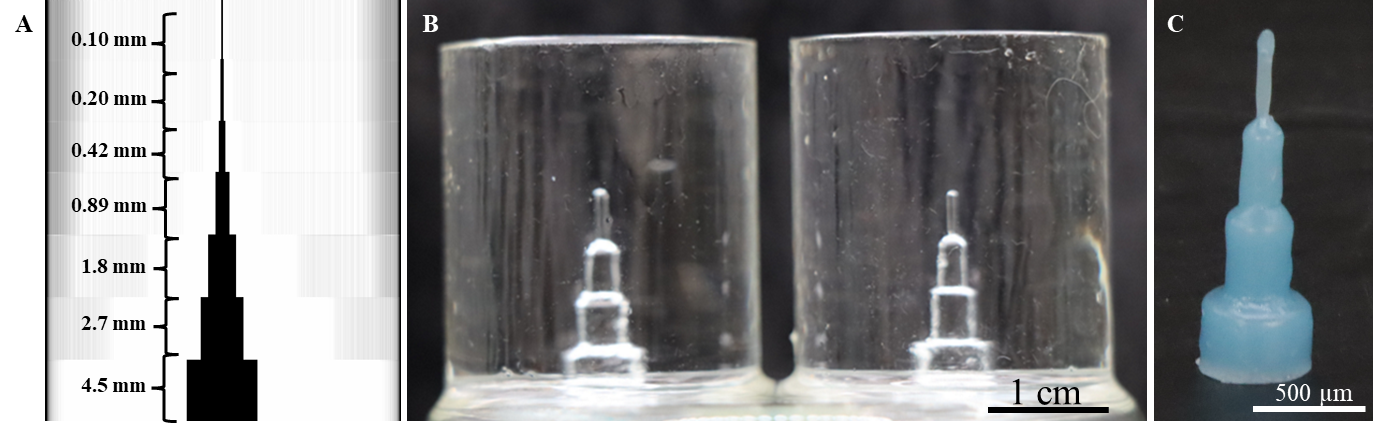


**Figure S3**. A) 3D model illustrating cylindrical stepwise resolution features with a range of diameters . B) LED-based VAM-printed resolution test demonstrating a minimum achievable resolution of approximately 1 mm. C) Extracted wax part from the cast resolution test.

| Projection Size (mm) | 4.5 | 2.7 | 1.8 | 0.89 | 0.42 | 0.20 | 0.10 |
| --- | --- | --- | --- | --- | --- | --- | --- |
| Average Mold Size (mm) | 5.1 | 3.0 | 1.8 | 0.58 | N/A | N/A | N/A |
| Standard Deviation | 0.14 | 0.12 | 0.11 | 0.13 | N/A | N/A | N/A |
| Average Percent Discrepancy of Mold to Projection Size (%) | 14 | 12 | -1 | -34 | N/A | N/A | N/A |

**Table S2**. Average wax mold size from the resolution tests compared to the projection size and the programmed STL.

| **Light Source** | **LED** | **Laser** |
| --- | --- | --- |
| DMD | 3DLP9000 | DLP9500 |
| Projection Optics | Telecentric lens | 4f System |
| Projected Pixel Size | 14.5 µm | 5.4 µm |
| Vial Inner Diameter | 2.5 cm | 1 cm |
| Maximum Intensity | 58 mW/cm^2^ | 1000 mW/cm^2^ |

**Table S3**. Differences between the LED and Laser VAM systems used to print the fluidic devices.


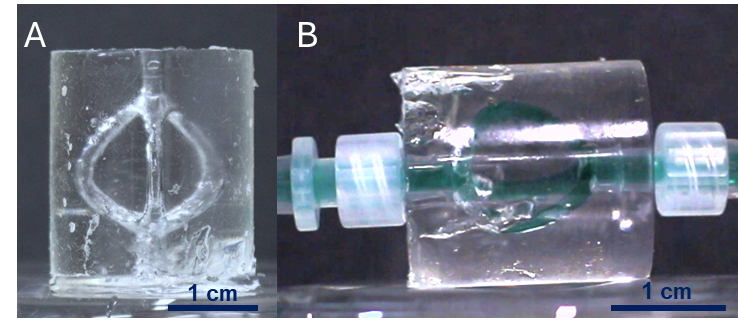


**Figure S4.** Example of VAM printed millifluidic devices without fluid (A) and with fluid (B) in the channels.

**
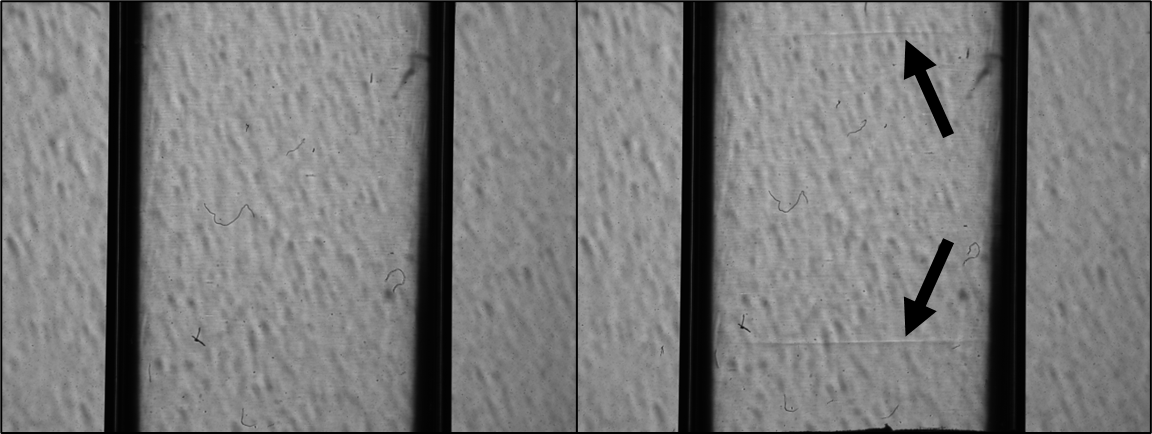
**

**Figure S5.** Before light exposure (left) and after light exposure (right) to form the microfluidic device in the laser VAM system. Arrows after exposure depict the top and bottom of the microfluidic device.


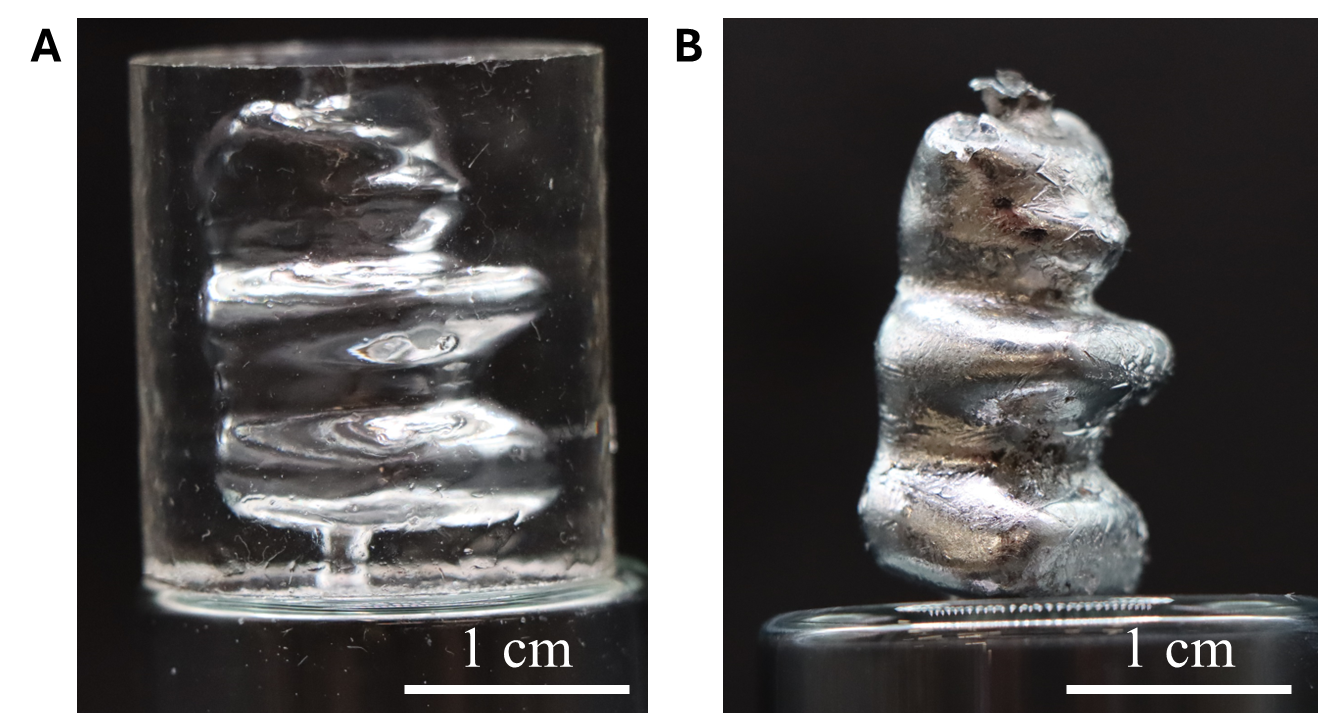


**Figure S6**. A) Printed bear instant mold after clearing the void of uncured resin. B) Extracted gallium bear cast from the printed instant mold.

**Mechanical Testing**

Printed dogbones exhibited lower elongations to break than molded samples (averaging 48% strain vs. 110% respectively), although they maintain similar elastomeric behavior **(Figure S7)**. The lowered elongations to break and ultimate tensile stresses are most likely attributed to outgrowth and deformations in the printed structures (more comparisons in **Table S3**). These non-uniform curing aspects often led the dogbones to fail near the clamps, which signifies defects in the bridge region of the dogbone. These curing non-uniformities are difficult to remove for the photohydrosilylation chemistry, as once the catalyst is activated it will continue to react, making timing essential for part resolution. Our ongoing and future efforts will be to improve the stability of the hydrosilylation chemistries through use of platinum catalysts poisons and quenching agents. While it is difficult to print positive features such as dogbones using traditional tomographic optimization strategies with good resolution, it is much simpler to keep channels and molds open, by not exposing certain regions of the container using a zero-dose approach.

**Figure S7.** Comparison of mechanical properties between printed (red hues) and molded bulk cast dogbones (black and grey hues).

| **Sample** | **Stress at 10% strain (kPa)** | **Ultimate Tensile Stress (MPa)** | **Strain to break (%)** |
| --- | --- | --- | --- |
| **Print 1** | 64.3 | 0.244 | 56 |
| **Print 2** | 63.4 | 0.176 | 33 |
| **Print 3** | 65.7 | 0.243 | 55 |
| **Average** | 64.5 | 0.221 | 48 |
| **StdDev** | 1.0 | 0.032 | 10.6 |
|  |  |  |  |
| **Cast 1** | 71.7 | 0.427 | 124 |
| **Cast 2** | 83.0 | 0.454 | 114 |
| **Cast 3** | 80.7 | 0.393 | 91.0 |
| **Average** | 78.5 | 0.425 | 109 |
| **StdDev** | 4.90 | 0.025 | 13.8 |
|  |  |  |  |
| **Difference (%)** | 17.8 | 48.0 | 56.2 |

**Table S4.** Comparison of silicone tensile dogbone samples.

| Sample | Millifluidic | Microfluidic |
| --- | --- | --- |
| Voltage (kV) | 60 | 50 |
| Current (µA) | 83 | 80 |
| Filter* | LE5 | LE3 |
| Source-to-rotation axis distance (mm) | 95.01 | 17.01 |
| Source-to-detector distance (mm) | 324.98 | 267.01 |
| Voxel size (µm) | 21.87 | 4.77 |
| Exposure (s) | 6 | 7 |
| Number of projections | 1601 | 3001 |
| Frames per projection | 20 | 20 |

**Table S5.** Acquisition settings for X-Ray CT scans.

| **Sample** | **Millifluidic** | **Microfluidic** |
| --- | --- | --- |
| Standard deviation of Gaussian smoothing filter σ (pixels) | 0.7 | 0.7 |
| Beam Hardening Correction coefficient | 0.11 | 0.25 |

**Table S6.** Tomographic reconstruction settings.

**Fluidic Channel Comparisons**

For the millifluidic channel size (Figure S8, Supplemental Information), the printed device has a smaller exterior channel compared to the other two STL models. On average, the interior channel of the printed device is similar to the reconstruction; however, it exhibits greater variation in diameter due to the spiral pattern. For the microfluidic channel (Figure S9, Supplemental Information), the reconstruction and CT scan STL models have larger diameters compared to the input STL. In terms of Z-axis thickness, the CT scan STL is more comparable to the input STL. However, the CT scan STL is smaller in the X and Y dimensions relative to the other two STL models.


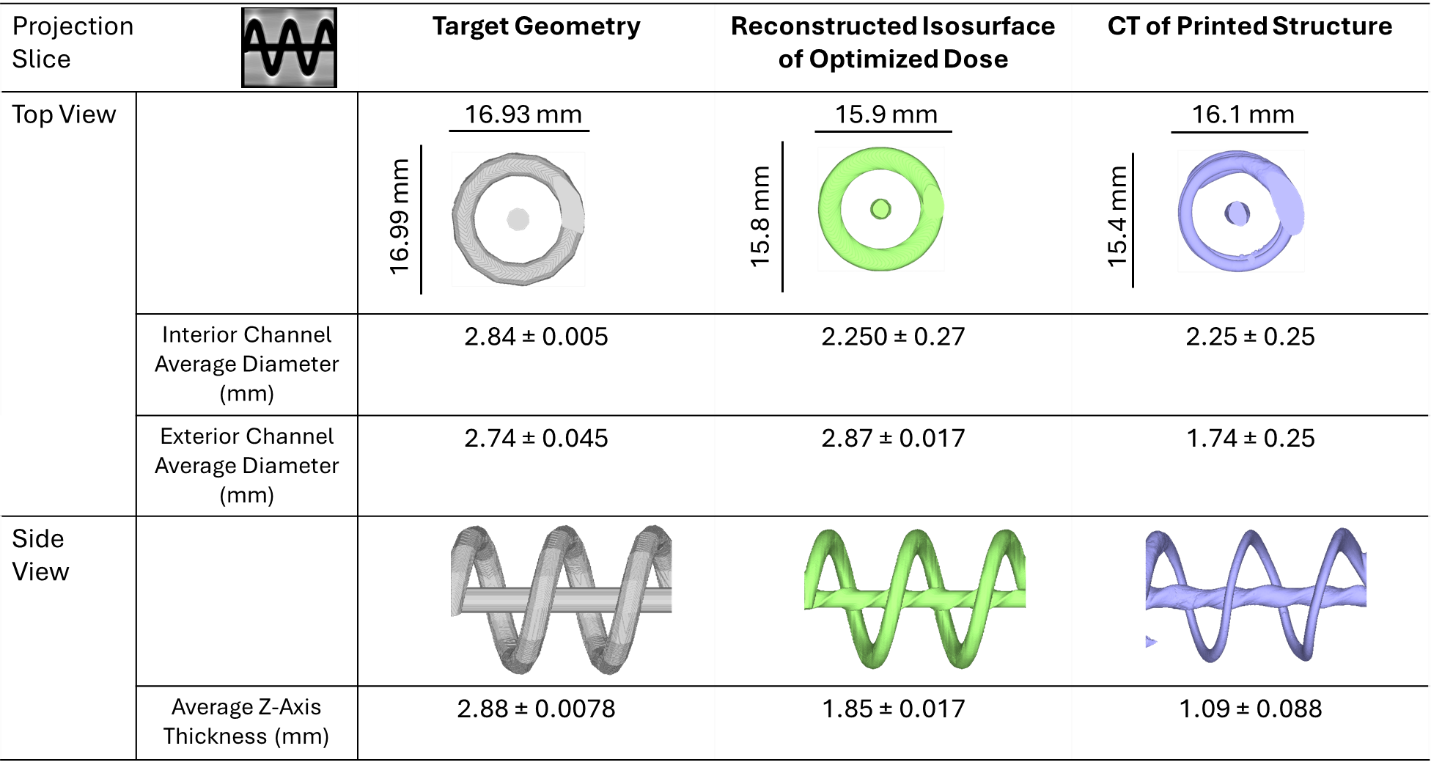


**Figure S8.** Millifluidic channel diameter and Z-axis thickness of the input target geometry compared to the reconstructed isosurface from the zero-dose optimization and the printed geometry generated from the CT scan. 3D STLs were all scaled to the CT STL Z height for the comparisons.


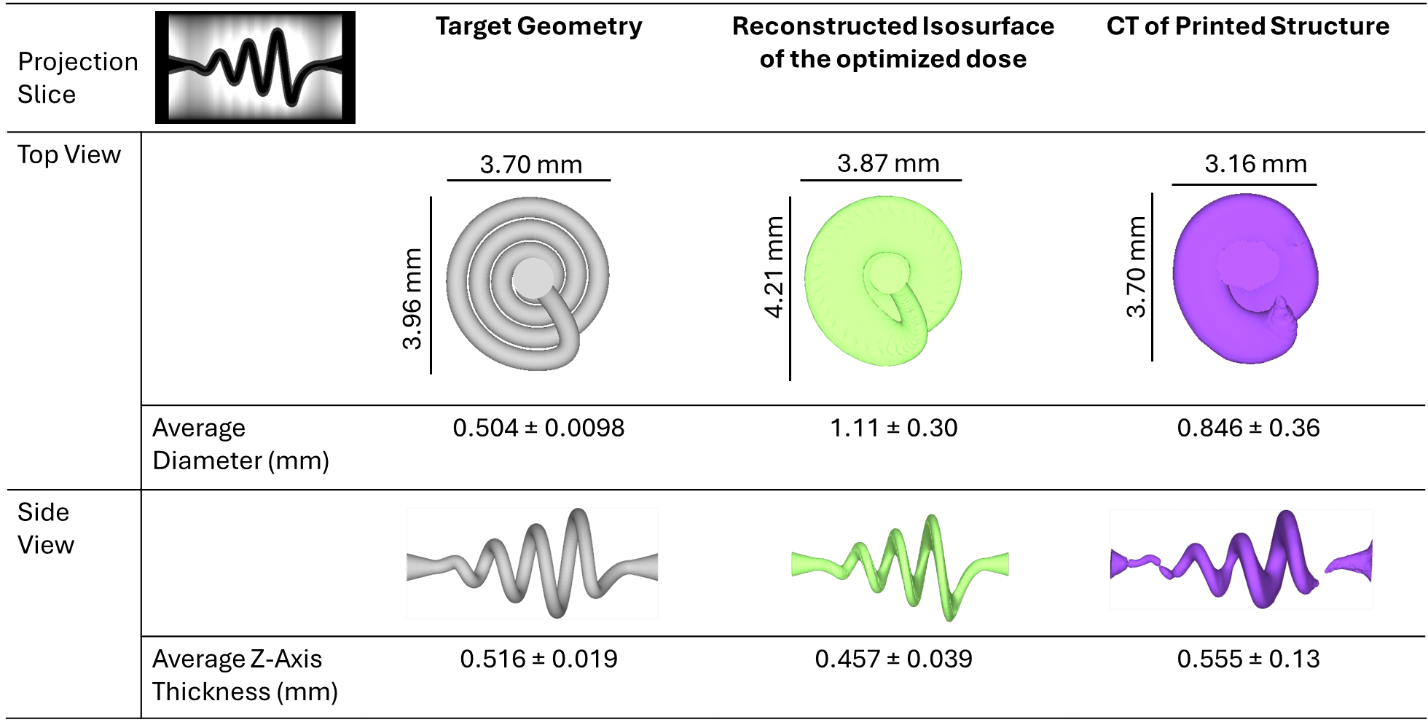


**Figure S9.** Microfluidic channel diameter and Z-axis thickness of the target geometry compared to the reconstructed isosurface from the zero-dose optimization and the printed geometry generated from the CT scan. 3D STLs were all scaled to the CT STL Z height for the comparisons.


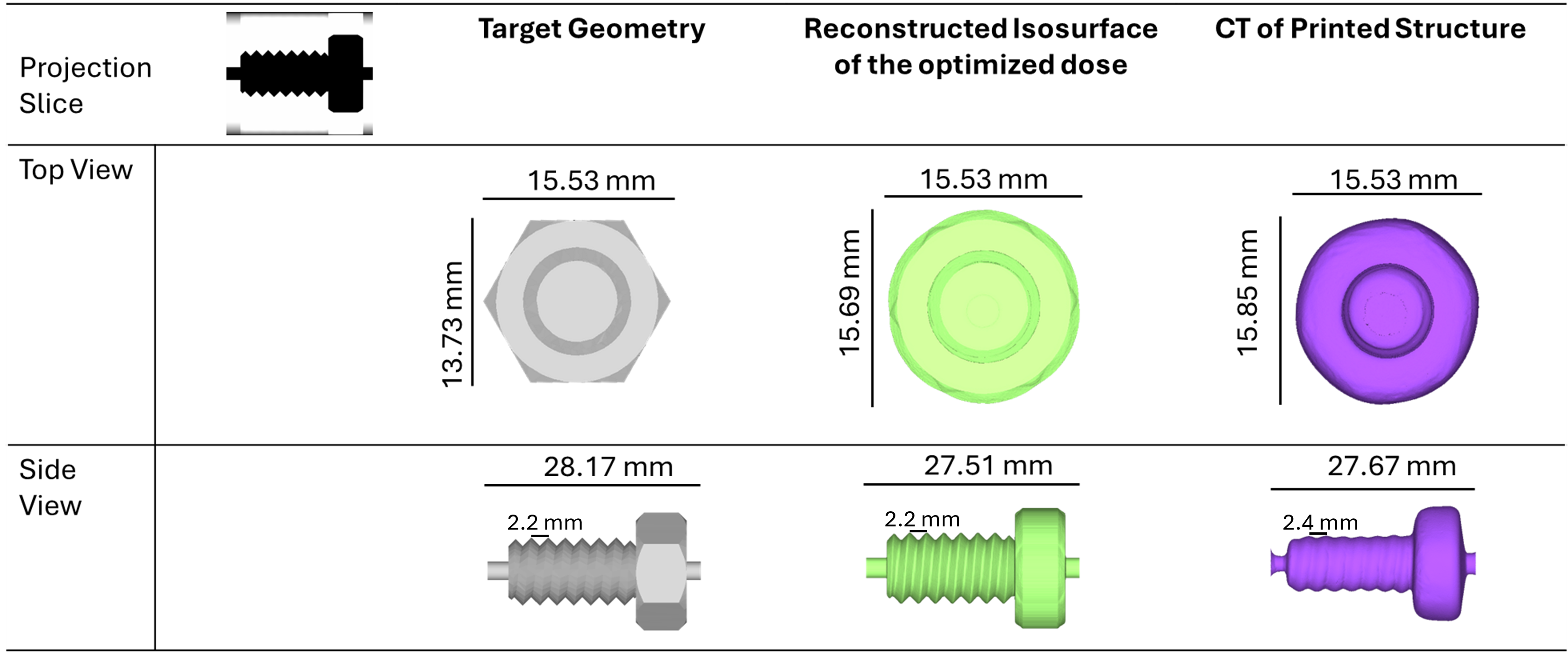


**Figure S10**. Instant screw mold size comparison of the input target geometry compared to the reconstructed isosurface and the printed geometry generated from the CT scan. The thread gap for the screws are on the side view, and the gaps are consistent with an M2 sized screw. 3D STLs were all scaled to the CT STL Z height of the screw (excluding the outlets) for the comparisons.


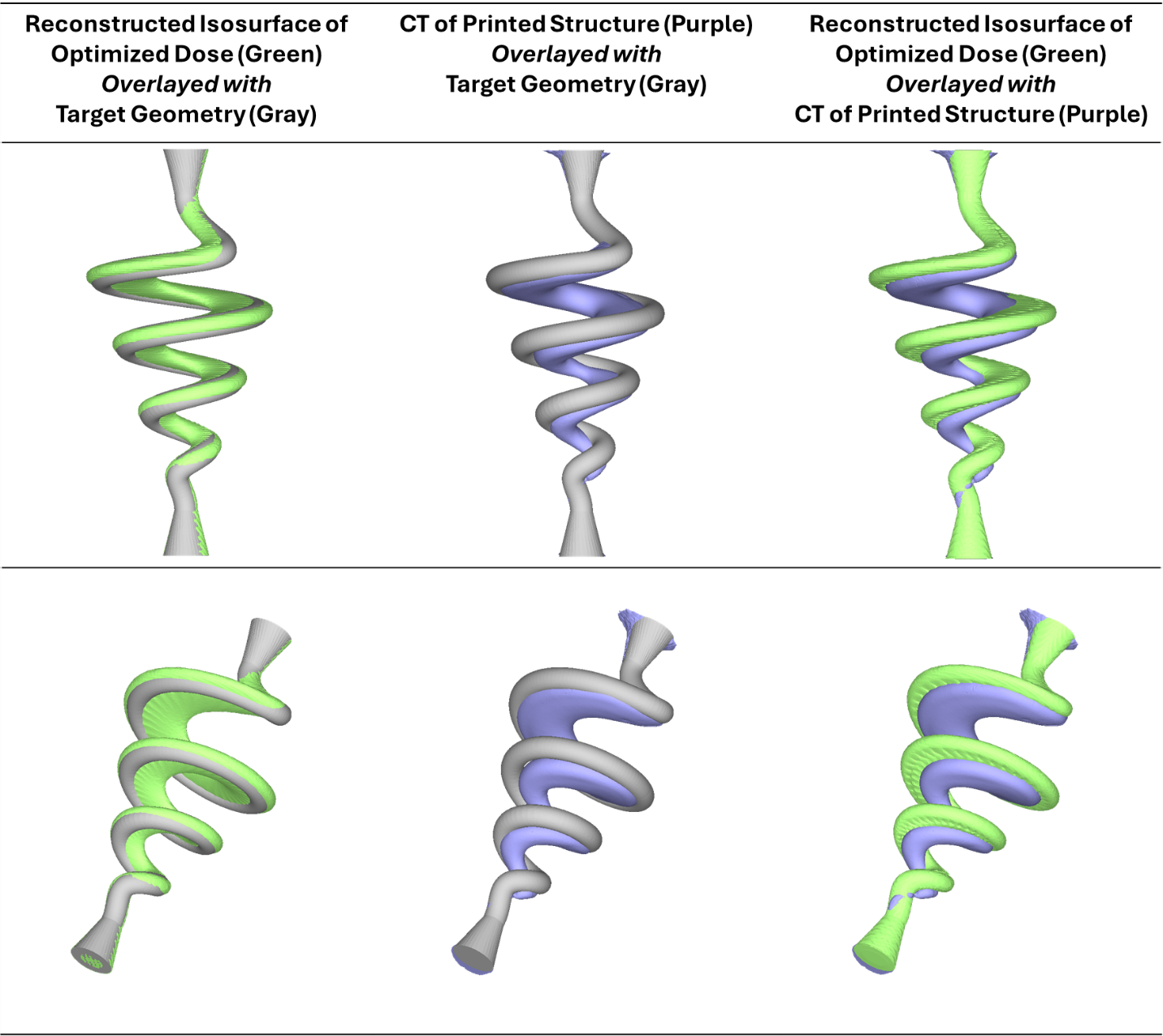


**Figure S11.** Comparisons between the input target geometry (gray) with the reconstructed isosurface from the zero-dose optimization (green) and the printed geometry from the CT scan of the VAM printed microfluidic device (purple).
